# Analgesia and sedation trends in a level IV NICU, 2014–2024: Opioid and dexmedetomidine use

**DOI:** 10.1038/s41372-026-02586-0

**Published:** 2026-02-23

**Authors:** Sin Yin Lim, Abrar Majeedi, Ryan M. McAdams

**Affiliations:** 1 Pharmacy Practice and Translational Research Division, School of Pharmacy, University of Wisconsin-Madison, Madison, WI USA; 2 Department of Biostatistics and Medical Informatics, School of Medicine and Public Health, University of Wisconsin-Madison, Madison, WI USA; 3 Department of Pediatrics, University of Wisconsin School of Medicine and Public Health, Madison, WI USA

**Keywords:** Paediatrics, Pain management

## Abstract

**Objective:**

Examine 11-year trends in opioid and sedative exposure and dosing practices in a level IV neonatal intensive care unit (NICU).

**Methods:**

This retrospective cohort study included NICU admissions from 2014 to 2024 that received at least one opioid or sedative. Temporal changes in patient characteristics, exposure patterns, continuous infusion regimen composition, and morphine and dexmedetomidine infusion rates were evaluated across three epochs (2014-2017, 2018-2021, 2022-2024).

**Results:**

Of 2055 admissions, 1060 (51.6%) encounters were analyzed. Infants admitted in later years were more premature with greater morbidity. Dexmedetomidine continuous infusion use increased fivefold (7.9% to 44.1%; *p* < 0.001), while morphine infusion use also increased (36.7% to 50.4%; *p* = 0.0021). Morphine plus dexmedetomidine became the most common continuous infusion regimen by 2022-2024 (28.7%), with higher infusion rates in combination therapy than in monotherapy.

**Conclusion:**

NICU sedation strategies shifted from morphine monotherapy toward combination therapy with dexmedetomidine. Standardized, outcome-oriented guidelines and multicenter studies are needed.

## Introduction

Critically ill neonates frequently undergo painful procedures during intensive care. Repetitive exposure to pain is associated with physiologic instability, abnormal brain maturation, and poorer neurodevelopment outcomes [1, 2]. Effective pain and sedation management in critically ill neonates is essential not only to facilitate necessary interventions, minimize stress, and improve patient comfort, but also to prevent long-term neurodevelopmental sequelae [3, 4]. Opioids, such as morphine and fentanyl, remain the cornerstone of analgesia, while sedatives, including benzodiazepines and α₂-adrenergic agonists, are used for anxiolysis and procedural tolerance [5]. However, optimal strategies in neonates, particularly those requiring continuous infusions, remain debated due to concerns about respiratory depression, tolerance, withdrawal, and neurodevelopmental impact [6, 7].

Despite decades of clinical use, no consensus exists on best practices for neonatal analgesia and sedation, and management strategies vary widely across institutions [8]. Over the past decade, dexmedetomidine—an α₂-adrenergic agonist with sedative and analgesic properties—has been increasingly adopted in neonatal care [9, 10]. Evidence suggests that dexmedetomidine provides effective sedation with minimal adverse effects and may reduce exposure to opioids and benzodiazepine [9, 11, 12]. While posing less risk of respiratory depression than opioids, its use in neonates remains largely off-label, and evidence on long-term safety is limited [13, 14].

The variability in practice patterns, coupled with limited comparative data and the evolving pharmacologic options, highlights the need to characterize real-world sedation trends in high-acuity neonatal intensive care units (NICUs). We therefore conducted an 11-year retrospective cohort study in a Level IV NICU to examine changes in opioid and sedative exposure, evaluate shifts in patient characteristics, and analyze dosing patterns, with particular focus on continuous infusion regimens of morphine and dexmedetomidine.

## Methods

### Study design and setting

We conducted a single-center retrospective cohort study in a 26-bed academic level IV NICU at the American Family Children’s Hospital, University of Wisconsin-Madison, Wisconsin, USA. The study was approved by the institutional review board with a waiver of informed consent.

### Population

All infants admitted between January 1, 2014, and December 31, 2024, who received at least one dose of an opioid or sedative were eligible for inclusion. Infants with multiple admissions during the study period were included as separate encounters. Infants were excluded if they had a corrected age >6 months at NICU admission, were admitted to the NICU for <50% of their total hospital stay, received palliative care, or were diagnosed with neonatal abstinence withdrawal syndrome.

### Data collection

Data were extracted from electronic medical records. Demographics included gestational age (GA), birth weight, sex, race/ethnicity, Apgar scores, postmenstrual age (PMA), and postnatal age (PNA) at admission. Clinical data included NICU and hospital length of stay, use of mechanical ventilation, mortality, and major diagnoses such as bronchopulmonary dysplasia, patent ductus arteriosus, and intraventricular hemorrhage, identified using ICD-10 codes (Supplementary Table S1).

Medication data included drug name, administration route, bolus versus continuous infusion (CI), start/stop times, and dosing details. The first therapy day was defined as the initial day during the hospital stay on which any evaluated opioid or sedative agent was administered. A list of the evaluated opioid and sedative agents is provided in Supplementary Table S2. Daily average infusion rates during the first seven days of therapy for morphine and dexmedetomidine CIs were calculated by dividing the total amount administered each day by 24 h, excluding as-needed boluses. Therapy transition phases were classified as initial (therapy days 1–3), intermediate (days 4–15), and late ( > day 15). Annual total NICU admissions were recorded for denominator calculations; no patient-specific data were collected for infants excluded by criteria.

### Outcomes

The primary outcome was temporal trends in opioid and sedative exposure among infants across epochs (2014–2017, 2018–2021, 2022–2024). Secondary outcomes included (1) changes in infant characteristics, (2) differences in composition of CI regimens, and (3) differences in morphine and dexmedetomidine dosing between monotherapy and combination therapy.

### Statistical analysis

We plotted yearly counts and percentages of NICU encounters involving study infants relative to total NICU admissions, assessing temporal trends with the Cochran-Armitage test. Among exposed infants, temporal changes in drug exposure, patient profiles, combination of CI regimens, and infusion rates of morphine and dexmedetomidine CIs were compared across epochs. Additionally, average daily infusion rates of morphine and dexmedetomidine CIs, when used alone versus in combination, were compared during the first week of therapy. To evaluate health equity among NICU patients, patient demographics were compared across different pain and sedation management strategies, based on whether the regimen included opioids or sedatives administered on a scheduled basis or as needed.

Continuous variables were reported as medians with interquartile ranges (IQR) and compared using t-tests or Mann–Whitney U tests for two-groups, and ANOVA or Kruskal–Wallis tests for more than two groups. Categorical variables were reported as frequencies with percentages, and compared with χ² or Fisher’s exact tests. Bonferroni correction was applied for multiple comparisons, as appropriate. Transitions in therapy over time were visualized using a Sankey flow diagram generated in RStudio (Posit team 2025; Posit Software, PBC, Boston, MA) and formatted in SankeyMATIC (https://sankeymatic.com/). Analyses were conducted using SAS version 9.4 (SAS Institute Inc., Cary, NC).

## Results

### Trends in agent use

Of the 2055 NICU admissions during the study period, 1060 encounters (51.6%) involved infants who received opioids or sedatives and met inclusion criteria (Supplementary Fig. S1). These encounters accounted for 39.1% to 63.8% of total NICU admissions across years, with no significant temporal trend observed (*p* = 0.86; Supplementary Fig. S2). Thirty-nine encounters (3.7%) involved repeated admissions of the same infants.

**Fig. 1:**
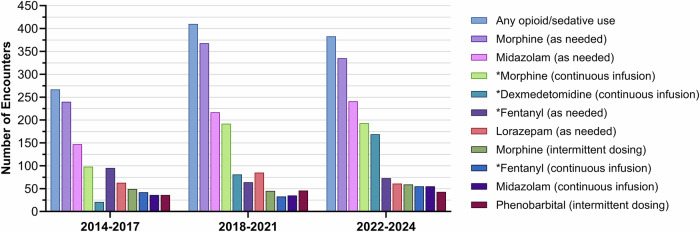
Number of encounters involving study infants who received any opioid or sedative agents, as well as those who received specific opioid or sedative agents. Asterisks in the figure legend indicate statistically significant differences across three epochs after Bonferroni correction (*p* < 0.005).

Across epochs, as-needed morphine bolus (87.5–89.9%) and midazolam bolus (52.9–62.9%) were the most frequently administered among the ten most commonly used agents, as shown in Fig. 1 and Supplementary Table S3. Dexmedetomidine CI use increased more than five-fold (7.9% in 2014-2017 to 44.1% in 2022-2024; *p* < 0.0001) and morphine CI increased from 36.7% to 50.4% (*p* = 0.0021). In contrast, fentanyl as-needed bolus use declined from 35.6% to 19.1% (*p* < 0.0001). The use of fentanyl CI (*p* = 0.0035) and midazolam CI (*p* = 0.0262) showed a fluctuating trend, decreasing from 14–16% in 2014–2017 to 8-8.5% in 2018–2021, then increasing to 14.4% in 2022–2024.

### Cohort characteristics

Infants in the 2022–2024 epoch were more premature and had higher morbidity compared with earlier epochs (Table 1). Median GA declined (36.4 vs. 37.1–37.3 weeks; *p* = 0.0064) and birth weight was lower (2.80 vs. 2.85-2.97 kg; *p* = 0.0044). The prevalence of several comorbidities increased over time, including patent ductus arteriosus (38.9% vs. 30.2–31.8%; *p* = 0.0268), congenital heart defects (54.6% vs. 44.1–48.3%, *p* = 0.0131), respiratory distress syndrome (32.9% vs. 16.5-27.6%; *p* < 0.0001), periventricular leukomalacia (3.4% vs. 0.7-1.2%; *p* = 0.0198), and positive culture of sterile sites (17% vs. 10.1–11.2%; *p* = 0.0146). When comparing different pain and sedation management strategies among all infants, as expected, those who received scheduled opioids and sedatives along with as-needed opioids and sedatives were the most premature and had the lowest birth weight, whereas those who did not receive scheduled sedatives were more mature and had higher birth weights. Sex, race, and ethnicity were similar across strategies (Supplementary Table S4).

**Table 1:** Patient demographics and clinical conditions.

	Median (IQR) or n (%)				
Variables^1^	Overall	2014-2017	2018-2021	2022-2024	P-value
Gestational age (week)	37 (32.07-39)	37.29 (32.86-39)	37.14 (33.43-39)	36.43 (30.71-38.57)	0.0064
Birth weight (kg)	2.69 (1.58-3.33)	2.64 (1.61-3.4)	2.86 (1.86-3.39)	2.55 (1.31-3.23)	0.0044
Weight at hospital admission (kg)	2.89 (2.19-3.5)	2.85 (2.16-3.54)	2.97 (2.35-3.46)	2.8 (2-3.51)	0.2558
Postmenstrual age at NICU admission (week)	38.43 (35.99-40.5)	38.64 (35.81-40.65)	38.62 (36.65-40.39)	38.06 (35.34-40.58)	0.7118
Postnatal age at NICU admission (day)	4.61 (1.08-24.66)	3.82 (1.28-20.66)	4.45 (1.12-21.91)	5.94 (0.89-31.58)	0.0613
Sex					0.8224
Female	484 (45.7)	119 (44.6)	192 (46.8)	173 (45.2)	
Male	576 (54.3)	148 (55.4)	218 (53.2)	210 (54.8)	
Race					0.7081
American Indian or Alaska Native	9 (0.8)	1 (0.4)	4 (1)	4 (1)	
Asian	37 (3.5)	11 (4.1)	13 (3.2)	13 (3.4)	
Black or African American	96 (9.1)	16 (6)	45 (11)	35 (9.1)	
Native Hawaiian or other Pacific Islander	3 (0.3)	1 (0.4)	1 (0.2)	1 (0.3)	
White	867 (81.8)	228 (85.4)	326 (79.5)	313 (81.7)	
Unknown	48 (4.5)	10 (3.7)	21 (5.1)	17 (4.4)	
Ethnic group					0.8672
Hispanic/Latino	94 (8.9)	23 (8.6)	37 (9)	34 (8.9)	
Not Hispanic or Latino	925 (87.3)	234 (87.6)	354 (86.3)	337 (88)	
Unknown	41 (3.9)	10 (3.7)	19 (4.6)	12 (3.1)	
Hospital length of stay (day)	21.94 (10.99-46.1)	24.97 (12-46.1)	20.33 (11.14-44.88)	21.86 (10.03-47.94)	0.4826
NICU length of stay (day)	20.54 (10.75-43.27)	23.8 (11.86-44.24)	19.37 (10.98-43.7)	20.11 (9.12-41.89)	0.6259
Percentage of time spent in NICU	99.73 (98.44-100)	99.51 (98.44-99.97)	99.74 (98.83-100)	100 (95.17-100)	0.0012
1-Min Apgar score	6 (3-8)	6 (3-8)	7 (3-8)	5 (2-8)	0.0045
5-Min Apgar score	8 (6-9)	8 (6-9)	8 (6-9)	8 (5-9)	0.182
10-Min Apgar score	7 (6-8)	7 (5-8)	7 (6-8)	7 (6-8)	0.4639
Mechanical ventilation	369 (34.8)	102 (38.2)	134 (32.7)	133 (34.7)	0.3375
ECMO use	0	0	0	0	NA
Procedure type^2^					0.2377
Invasive	317 (29.9)	89 (33.3)	118 (28.8)	110 (28.7)	
Minimally Invasive	95 (9)	24 (9)	35 (8.5)	36 (9.4)	
Noninvasive	15 (1.4)	7 (2.6)	5 (1.2)	3 (0.8)	
None	633 (59.7)	147 (55.1)	252 (61.5)	234 (61.1)	
Gastroschisis	39 (3.7)	15 (5.6)	14 (3.4)	10 (2.6)	0.1258
Omphalocele	16 (1.5)	2 (0.7)	4 (1)	10 (2.6)	0.0832
Congenital diaphragmatic hernia	37 (3.5)	9 (3.4)	11 (2.7)	17 (4.4)	0.4011
Necrotizing enterocolitis	45 (4.2)	10 (3.7)	16 (3.9)	19 (5)	0.6821
Intestinal perforation	30 (2.8)	9 (3.4)	10 (2.4)	11 (2.9)	0.7733
Bronchopulmonary dysplasia	219 (20.7)	54 (20.2)	77 (18.8)	88 (23)	0.3382
Congenital malformation of the lung	37 (3.5)	11 (4.1)	14 (3.4)	12 (3.1)	0.7921
Respiratory distress syndrome	283 (26.7)	44 (16.5)	113 (27.6)	126 (32.9)	<0.0001
Apnea of prematurity	261 (24.6)	63 (23.6)	100 (24.4)	98 (25.6)	0.837
Persistent pulmonary hypertension of the newborn	103 (9.7)	1 (0.4)	42 (10.2)	60 (15.7)	<0.0001
Meconium aspiration syndrome	23 (2.2)	7 (2.6)	12 (2.9)	4 (1)	0.1579
Cystic fibrosis	7 (0.7)	6 (2.2)	1 (0.2)	0 (0)	0.0013
Neonatal seizure	102 (9.6)	21 (7.9)	46 (11.2)	35 (9.1)	0.324
Neonatal stroke	12 (1.1)	0 (0)	7 (1.7)	5 (1.3)	0.1075
Hypoxic ischemic encephalopathy	112 (10.6)	22 (8.2)	43 (10.5)	47 (12.3)	0.2579
Periventricular leukomalacia	20 (1.9)	2 (0.7)	5 (1.2)	13 (3.4)	0.0198
Congenital hydrocephalus	53 (5)	9 (3.4)	25 (6.1)	19 (5)	0.2818
Intraventricular hemorrhage, grade 1 or 2	48 (4.5)	9 (3.4)	18 (4.4)	21 (5.5)	0.4376
Intraventricular hemorrhage, grade 3 or 4	43 (4.1)	13 (4.9)	12 (2.9)	18 (4.7)	0.3322
Intraventricular hemorrhage, unspecified	19 (1.8)	4 (1.5)	8 (2)	7 (1.8)	0.9176
Patent ductus arteriosus	358 (33.8)	85 (31.8)	124 (30.2)	149 (38.9)	0.0268
Congenital heart defect	519 (49)	129 (48.3)	181 (44.1)	209 (54.6)	0.0131
Acute kidney injury	99 (9.3)	20 (7.5)	38 (9.3)	41 (10.7)	0.3822
Renal dysplasia	15 (1.4)	5 (1.9)	5 (1.2)	5 (1.3)	0.7611
Congenital nephrotic syndrome	0	0	0	0	NA
Renal agenesis and other reduction defects	6 (0.6)	2 (0.7)	1 (0.2)	3 (0.8)	0.5885
Anemia of prematurity	172 (16.2)	45 (16.9)	58 (14.1)	69 (18)	0.3191
Positive culture of sterile sites	138 (13)	27 (10.1)	46 (11.2)	65 (17)	0.0146
Bacterial meningitis	17 (1.6)	5 (1.9)	4 (1)	8 (2.1)	0.4278
Urinary tract infection	25 (2.4)	5 (1.9)	9 (2.2)	11 (2.9)	0.6839
Bacterial sepsis	91 (8.6)	28 (10.5)	32 (7.8)	31 (8.1)	0.4347
Died	67 (6.3)	18 (6.7)	23 (5.6)	26 (6.8)	0.7515

*IQR* interquartile range, *n* frequency, *NICU* neonatal intensive care unit, *ECMO* extracorporeal membrane oxygenation, *NA* not applicable.

^1^Number of missing values: birth weight=14, weight at hospital admission=1, 1-min Apgar=188, 5-min Apgar=186, 10-min Apgar=708.

^2^Procedure invasiveness was defined based on whether the procedure involved intentional penetration of the skin or mucosa, or entry into body cavities or internal organs (invasive); access to internal structures via small incisions or natural orifices (minimally invasive); or neither (noninvasive).

### Continuous infusion regimens and therapy transitions

**Fig. 2:**
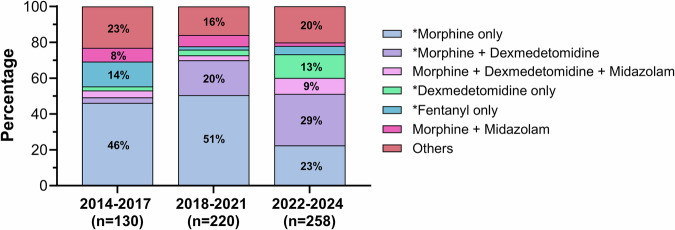
Changes in percentages of various continuous-infusion regimens over time. Asterisks in the figure legend represent statistically significant differences across three epochs after Bonferroni correction (*p* < 0.007).

**Fig. 3:**
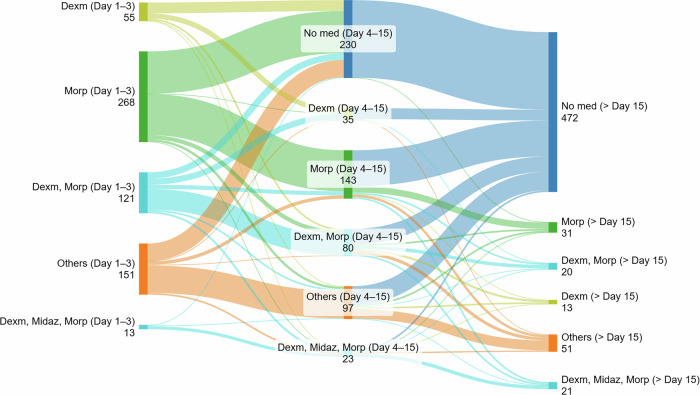
Sankey diagram illustrating transitions between continuous-infusion regimens during the initial (Days 1-3), intermediate (Days 4-15), and late ( > Day 15) treatment phases.

The proportion of encounters with infants receiving any CI increased from 48.7% in 2014–2017 to 67.4% in 2022–2024 (*p* < 0.0001). Among 608 encounters with infants on CIs, morphine monotherapy decreased from 46.2% in 2014–2017 and 50.5% in 2018–2021 to 22.5% in 2022–2024 (*p* < 0.0001; Fig. 2). In contrast, the combination of morphine and dexmedetomidine became the most common regimen, increasing from 3.1% in 2014-2017 to 28.7% in 2022-2024 (*p* < 0.0001). Dexmedetomidine monotherapy also increased from 2.4% to 13.2% (*p* < 0.0001). The use of morphine plus midazolam decreased from 7.7% to 1.9% (*p* = 0.0163), while triple therapy with morphine, dexmedetomidine, and midazolam increased from 3.8% to 8.9% (*p* = 0.0084). Most infants remained on their initial regimen or de-escalated to no infusion from initial (days 1–3) to intermediate (days 4–15) phase (Fig. 3). From the intermediate to late ( > day 15) phase, most de-escalated to no infusion, and escalation from monotherapy to combination regimens was uncommon.

### Dosing patterns of morphine and dexmedetomidine continuous infusions

**Fig. 4:**
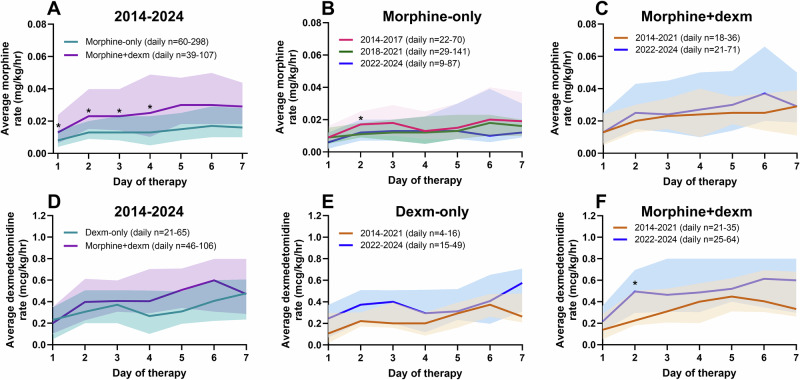
Average infusion rates of morphine and dexmedetomidine continuous infusions during the first seven days of therapy, administered either as monotherapy or in combination. **A–C** display average morphine infusion rates: combined across 2014–2024 (**A**), and stratified by time periods **B, C**. **D–F** show average dexmedetomidine infusion rates: combined across 2014–2024 (**D**), and stratified by time periods **E, F**. Asterisks indicate statistically significant differences in daily infusion rates after Bonferroni correction (*p* < 0.007).

Average daily infusion rates for morphine were significantly higher when administered together with dexmedetomidine than when used as monotherapy (p < 0.007; Fig. 4). Dexmedetomidine infusion rates increased in 2022–2024 compared with earlier epochs, and tended to be higher when administered in combination with morphine.

## Discussion

In this 11-year single-center study, we observed a clear shift in analgesia and sedation practices toward combination therapy, particularly morphine plus dexmedetomidine. Morphine monotherapy declined substantially, while dexmedetomidine, initially introduced as an opioid-sparing alternative, was increasingly administered as an adjunct rather than a replacement. Combination therapy was associated with higher infusion rates of both agents, and these changes occurred alongside rising infant acuity, including lower GA, reduced birth weight, and higher rates of respiratory distress syndrome and congenital heart defects.

Dexmedetomidine use among study infants increased fivefold, from 7.9% in 2014–2017 to 44.1% in 2022–2024, representing the largest shift in sedation practice. By 2022–2024, dexmedetomidine was most often given with morphine (28.7%), less commonly as monotherapy (13.2%), or in triple therapy with morphine and midazolam (8.9%). Our findings align with prior studies demonstrating growing use of dexmedetomidine in neonatal and pediatric intensive care. Chrysostomou et al. reported in a Phase II/III trial [9]. that dexmedetomidine provided effective sedation without major adverse effects in preterm and term infants. Whalen et al. [11] demonstrated that adding three or more days of dexmedetomidine infusion to opioid and benzodiazepine improved comfort and sedation in critically ill children and infants, while reducing exposure to opioids or benzodiazepines.

More recently, dexmedetomidine use has expanded to include sedation during therapeutic hypothermia in infants with hypoxic-ischemic encephalopathy, as it also inhibits shivering and may potentially provide neuroprotective effects, as shown in numerous animal models [15–17]. Similarly, its use has expanded to include very preterm and extremely preterm infants, with no serious adverse effects or long-term neurodevelopmental outcomes observed at 6 months to 2 years of age [18–20]. However, since these studies were observational and had small sample sizes, the safety of dexmedetomidine in neonates needs to be confirmed. Our data add to this literature by showing how dexmedetomidine has become embedded in practice over a decade, not only as monotherapy but more commonly in combination regimens.

We observed a significant increase in the use of continuous-infusion regimens use over the three epochs (from 48.7% in 2014–2017, to 53.6% in 2018–2021, and 67.4% in 2022–2024). The increased reliance on combination therapy may reflect several factors. Higher acuity in recent years likely contributed, as sicker infants often require deeper or more sustained sedation. Variations in patient acuity may influence the number of painful procedures, the complexity of surgeries, and the invasiveness of respiratory support required, leading to different needs for analgesia and sedation [21]. Institutional factors, such as evolving order sets, nursing comfort, or availability of agents, may also influence prescribing. At out institution, pain management-related guidelines involving neonates were published internally in 2017 and 2022. The 2017 guideline did not identify specific opioid or sedative agents as preferred therapy for major procedures and postoperative pain in pediatric and neonatal patients. However, the 2022 guideline for postoperative pain management in the NICU specifies that morphine is the recommended opioid agent, while dexmedetomidine and midazolam are recommended as the second- and third-line agents, respectively, when escalation is needed. The updates in the 2022 institutional practice guideline may reflect evolving practices from prior years (2018–2021) and likely contributed to prescribing patterns observed in 2022–2024. The finding that both morphine and dexmedetomidine average daily infusion rates were higher in combination than monotherapy suggests possible confounding by indication: infants requiring combination therapy were likely more ill, with greater sedation needs. However, this pattern raises important safety questions, given that our observed dexmedetomidine doses in combination regimens exceeded ranges reported in early trials [9].

Our study also highlights changing roles of other agents. Use of fentanyl monotherapy infusions declined, perhaps reflecting a change in preference for opioid agents. Although fentanyl has a rapid onset of action and a shorter half-life compared with morphine, it may also have a greater risk for opioid tolerance and withdrawal following prolonged use [22]. Midazolam infusion use decreased when combined with morphine but increased in triple regimens with morphine and dexmedetomidine. This may reflect the role of dexmedetomidine in reducing exposure and dose requirement of midazolam, since midazolam has been associated with neurodevelopmental risks in neonates [23, 24]. Morton et al. showed that implementing sedation guidelines in a NICU increased the initiation of dexmedetomidine, which in turn reduced the use of midazolam by 30%, increased midazolam-free days from 0.3 to 2.2 days, and lowered midazolam dosages from 2.2 mg/kg/day to 1.3 mg/kg/day [25]. Interestingly, we also observed that infants who received triple therapy—morphine, dexmedetomidine, and midazolam—often remained on the regimen for a prolonged period ( > 15 days). This subset of infants may warrant closer attention, as prolonged exposure to both opioids and benzodiazepines ( > 7 days of therapy) in the NICU among extremely preterm neonates has been associated with poorer neurodevelopmental outcomes at 2 years of age [26].

### Strengths and limitations

Strengths of this study include a large cohort, decade-long timeframe, and detailed dosing analysis, which together provide a comprehensive picture of evolving NICU sedation practice. Study limitations include its retrospective, single-center design, which may limit generalizability. This study did not evaluate sedation scores, extubation outcomes, withdrawal, or long-term neurodevelopment, so the clinical impact of these shifts remains uncertain. Additionally, while opioids and sedatives are central, non-opioid analgesics and non-pharmacologic interventions remain critical adjuncts and were not captured in this study. For example, postoperative use of intravenous acetaminophen in neonates and infants has been shown to reduce opioid exposure while achieving similar pain relief [27, 28]. The use of intravenous acetaminophen at our institution may influence the observed trends of opioid use. Exposure classification may have been affected by overlapping infusions, and residual confounding is possible. As this study was conducted in a single high-acuity U.S. NICU, the findings may not reflect practice patterns in lower-acuity units or in resource-limited settings.

## Conclusion

Over a decade, sedation practices in this Level IV NICU evolved from morphine monotherapy toward morphine–dexmedetomidine combination therapy, accompanied by higher infusion rates and increased patient acuity. While combination therapy may offer sedation depth or stability, it raises important questions about cumulative drug exposure, tolerance, withdrawal risk, and long-term neurodevelopmental effects [29]. These findings underscore the need for standardized, evidence-based guidelines to balance efficacy with safety. Future multicenter studies should not only track adoption patterns but also evaluate short-term outcomes such as withdrawal, extubation readiness, and hemodynamic stability; long-term neurodevelopment, growth, and rehospitalization; and the impact of implementing standardized sedation guidelines on patient outcomes.

## Supplementary information

Supplemental material

## Data Availability

Data collected are available upon request from the corresponding author.
