# Supplementary material for: Analgesia and sedation trends in a level IV NICU, 2014–2024: Opioid and dexmedetomidine use
Source: J Perinatol. 2026 Feb 23;46(4):605–11. doi: 10.1038/s41372-026-02586-0 (PMC13121017; doi:10.1038/s41372-026-02586-0)
Supplement: Supplementary file 1 — Supplemental material [file 41372_2026_2586_MOESM1_ESM.docx]

**Supplementary Material**

**Analgesia and Sedation Trends in a Level IV NICU, 2014–2024: Opioid and Dexmedetomidine Use**

**Running title**: NICU Analgesia–Sedation Trends, 2014–2024

**Authors**: Sin Yin Lim^1,*^; Abrar Majeedi^2^; Ryan M. McAdams^3^

**Affiliations**: ^1^Pharmacy Practice and Translational Research Division, School of Pharmacy, University of Wisconsin-Madison, Madison, WI, ^2^ Department of Biostatistics and Medical Informatics, School of Medicine and Public Health, University of Wisconsin-Madison, Madison, WI, ^3^Department of Pediatrics, University of Wisconsin School of Medicine and Public Health, Madison, WI

***Corresponding author:**

Sin Yin Lim

777 Highland Ave

Madison, WI 53705

Sinyin.lim@wisc.edu

**Table S1**. List of ICD-10 codes of clinical diagnoses.

| **Diagnosis** | **ICD 10 Code** |
| --- | --- |
| Acute kidney injury | N17* |
| Anemia of prematurity | P61.2 |
| Apnea of prematurity | P28.4* |
| Bacterial meningitis | G00* |
| Bronchopulmonary dysplasia | P27.1, P27.9, P28.9, P28.89, or J98.4 |
| Congenital diaphragmatic hernia | Q79.0 |
| Congenital heart defect | Q20-Q24.9 |
| Congenital hydrocephalus | Q03* |
| Congenital malformation of lung | Q33* |
| Congenital nephrotic syndrome | N04.9 |
| Cystic fibrosis | E84* |
| Gastroschisis | Q79.3 |
| Grade 1 or 2 Intraventricular Hemorrhage | P52.0, P52.1 |
| Grade 3 or 4 Intraventricular Hemorrhage | P52.2* |
| Hypoxic ischemic encephalopathy | P91.6* |
| Intestinal perforation | P78.0 |
| Meconium aspiration syndrome | P24.01 |
| Necrotizing enterocolitis | P77* |
| Bacterial sepsis | P36* |
| Neonatal seizures | P90 |
| Neonatal stroke | P91.82* |
| Urinary tract infection | P39.3 |
| Neonatal withdrawal symptoms from maternal drug use | P96.1 |
| Omphalocele | Q79.2 |
| Patent ductus arteriosus | Q25.0 |
| Periventricular leukomalacia | P91.2 |
| Persistent pulmonary hypertension of the newborn | P29.30 |
| Renal agenesis or other reduction defects | Q60* |
| Renal dysplasia | Q61.4 |
| Respiratory distress syndrome | P22* |
| Retinopathy of prematurity | H35.1-H35.169 |
| Unspecified Intraventricular Hemorrhage | P52.3 |

*Included its extensions

**Table S2**. List of opioid and sedative agents evaluated in the study.

| **Opioids** | **Sedatives** |
| --- | --- |
| Fentanyl | Clonidine |
| Hydromorphone | Dexmedetomidine |
| Methadone | Diazepam |
| Morphine | Etomidate |
| Oxycodone | Gabapentin |
|  | Ketamine |
|  | Lorazepam |
|  | Midazolam |
|  | Pentobarbital |
|  | Phenobarbital |
|  | Propofol |

**Table S3**. Ten most commonly used opioid or sedative agents.

| **Medication** | **2014-2017 (n=267)** | **2018-2021 (n=410)** | **2022-2024 (n=383)** | **p-value** |
| --- | --- | --- | --- | --- |
| Morphine (as needed) | 240 (89.9) | 368 (89.8) | 335 (87.5) | 0.5047 |
| Midazolam (as needed) | 147 (55.1) | 217 (52.9) | 241 (62.9) | 0.0131 |
| Morphine (continuous infusion) | 98 (36.7) | 192 (46.8) | 193 (50.4) | 0.0021 |
| Dexmedetomidine (continuous infusion) | 21 (7.9) | 81 (19.8) | 169 (44.1) | <.0001 |
| Fentanyl (as needed) | 95 (35.6) | 64 (15.6) | 73 (19.1) | <.0001 |
| Lorazepam (as needed) | 63 (23.6) | 85 (20.7) | 61 (15.9) | 0.0433 |
| Morphine (intermittent dosing) | 49 (18.4) | 45 (11) | 59 (15.4) | 0.0226 |
| Fentanyl (continuous infusion) | 42 (15.7) | 33 (8) | 55 (14.4) | 0.0035 |
| Midazolam (continuous infusion) | 36 (13.5) | 35 (8.5) | 55 (14.4) | 0.0262 |
| Phenobarbital (intermittent dosing) | 36 (13.5) | 46 (11.2) | 43 (11.2) | 0.6124 |

**Table S4**. Patient demographics among infants who received different pain and sedation management strategies^1^.

|  | **Median (IQR) or n (%)** | | | | | | |  |
| --- | --- | --- | --- | --- | --- | --- | --- | --- |
| **Variables** | **Regimen 1** | **Regimen 2** | **Regimen 3** | **Regimen 4** | **Regimen 5** | **Regimen 6** | **Others** | **P-value** |
| Gestational age (week) | 35.57 (28-38.57) | 36.57 (31.71-38.86) | 36.64 (31.86-38.86) | 37.29 (35.43-38.57) | 38.14 (35-39.14) | 37.14 (32.43-39.14) | 38.14 (36-39.29) | <.0001 |
| Birth weight (kg) | 2.36 (0.98-3.17) | 2.55 (1.57-3.35) | 2.44 (1.43-3.18) | 2.8 (2.08-3.39) | 2.93 (2.23-3.44) | 2.87 (1.49-3.43) | 3.04 (2.42-3.46) | <.0001 |
| Weight at hospital admission (kg) | 2.8 (1.87-3.39) | 3.13 (2.4-3.97) | 2.47 (1.85-3.21) | 2.77 (2.35-3.43) | 3 (2.32-3.42) | 2.99 (2.1-3.48) | 3.2 (2.66-3.71) | <.0001 |
| Postmenstrual age at NICU admission (week) | 37.5 (34.12-40.35) | 39.4 (37.28-42.25) | 37.43 (33.78-39.36) | 37.83 (36.32-39.91) | 38.71 (36.77-39.94) | 38.86 (36.65-40.52) | 39.09 (37.33-40.95) | <.0001 |
| Postnatal age at NICU admission (day) | 6.57 (1.02-31.93) | 14.9 (2.46-56.59) | 1.73 (0.9-12.9) | 3.74 (1.43-13.59) | 1.6 (0.71-8.98) | 9 (1.56-21.53) | 4.64 (1.07-17.85) | <.0001 |
| Sex |  |  |  |  |  |  |  | 0.3685 |
| Female | 128 (46.5) | 97 (45.5) | 83 (53.2) | 54 (41.2) | 36 (39.6) | 27 (47.4) | 59 (43.1) |  |
| Male | 147 (53.5) | 116 (54.5) | 73 (46.8) | 77 (58.8) | 55 (60.4) | 30 (52.6) | 78 (56.9) |  |
| Race |  |  |  |  |  |  |  | 0.349 |
| American Indian or Alaska Native | 1 (0.4) | 3 (1.4) | 3 (1.9) | 0 (0) | 0 (0) | 2 (3.5) | 0 (0) |  |
| Asian | 13 (4.7) | 9 (4.2) | 3 (1.9) | 3 (2.3) | 1 (1.1) | 4 (7) | 4 (2.9) |  |
| Black or African American | 26 (9.5) | 21 (9.9) | 12 (7.7) | 9 (6.9) | 7 (7.7) | 5 (8.8) | 16 (11.7) |  |
| Native Hawaiian or other Pacific Islander | 0 (0) | 0 (0) | 2 (1.3) | 1 (0.8) | 0 (0) | 0 (0) | 0 (0) |  |
| White | 220 (80) | 173 (81.2) | 130 (83.3) | 111 (84.7) | 78 (85.7) | 44 (77.2) | 111 (81) |  |
| Unknown | 15 (5.5) | 7 (3.3) | 6 (3.8) | 7 (5.3) | 5 (5.5) | 2 (3.5) | 6 (4.4) |  |
| Ethnic group |  |  |  |  |  |  |  | 0.6132 |
| Hispanic/Latino | 28 (10.2) | 17 (8) | 18 (11.5) | 12 (9.2) | 4 (4.4) | 5 (8.8) | 10 (7.3) |  |
| Not Hispanic or Latino | 234 (85.1) | 191 (89.7) | 134 (85.9) | 113 (86.3) | 81 (89) | 51 (89.5) | 121 (88.3) |  |
| Unknown | 13 (4.7) | 5 (2.3) | 4 (2.6) | 6 (4.6) | 6 (6.6) | 1 (1.8) | 6 (4.4) |  |

^1^Regimen 1: scheduled opioids, scheduled sedatives, as-needed opioids, and as-needed sedatives; Regimen 2: as-needed opioids only; Regimen 3: scheduled opioids, as-needed opioids, and as-needed sedatives; Regimen 4: as-needed opioids and as-needed sedatives; Regimen 5: scheduled opioids and as-needed opioids; Regimen 6: scheduled sedatives, as-needed opioids, and as-needed sedatives; Other: all other combinations.


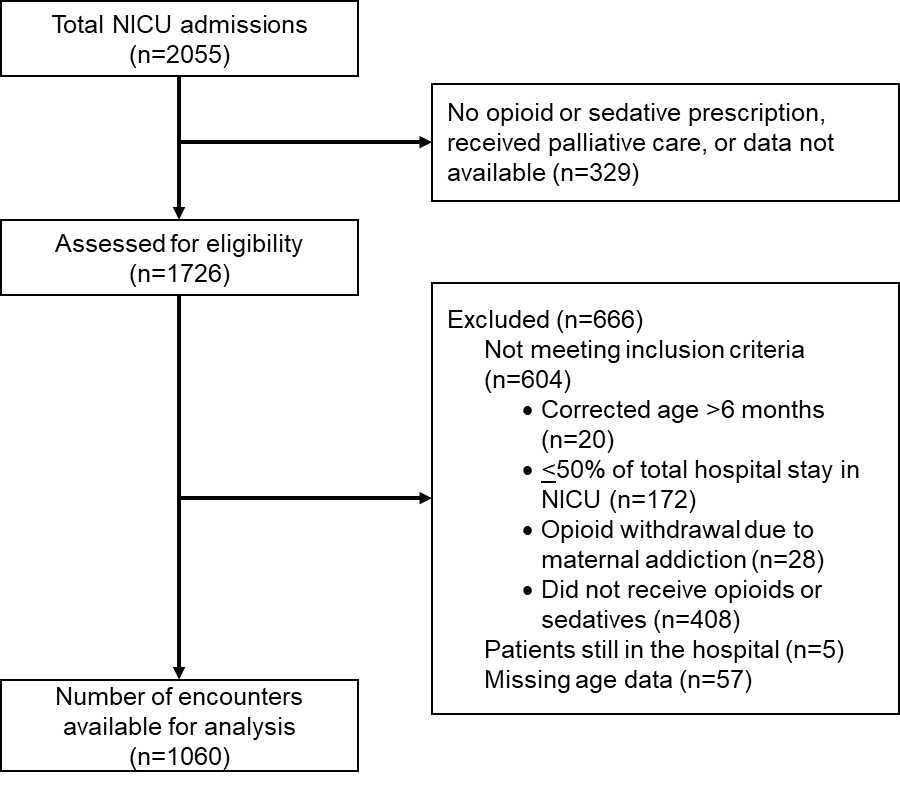


**Figure S1**. Patient flow diagram.


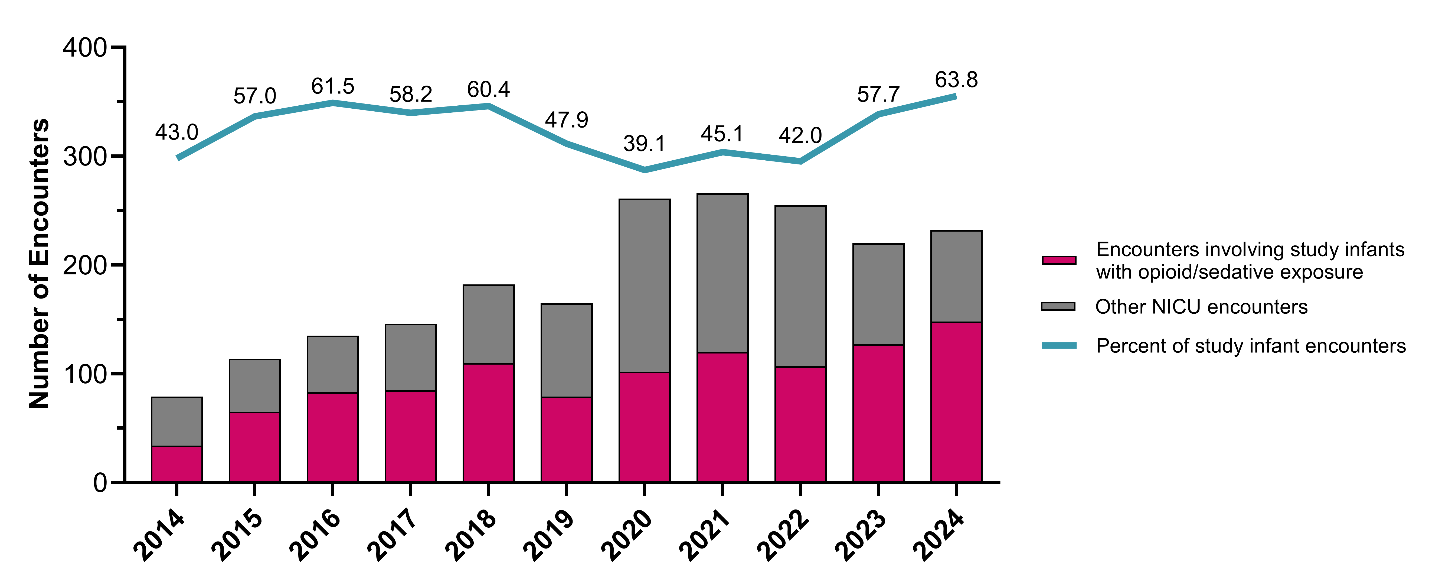


**Figure S2**. Number of all NICU admissions and the number and percentage of encounters involving study infants from 2014 to 2024; p=0.86 by Cochran-Armitage test for trend.
